# Cystatin C vs creatinine eGFR in advanced CKD: an analysis of the STOP-ACEi trial

**DOI:** 10.1093/ckj/sfae268

**Published:** 2024-08-29

**Authors:** Sebastian Spencer, Robert Desborough, Samir Mehta, Natalie Ives, Sunil Bhandari

**Affiliations:** University of Hull; Hull York Medical School; Hull University Teaching Hospitals NHS Trust; Hull York Medical School; Hull University Teaching Hospitals NHS Trust; Birmingham Clinical Trials Unit, University of Birmingham; Birmingham Clinical Trials Unit, University of Birmingham; Hull York Medical School; Hull University Teaching Hospitals NHS Trust

**Keywords:** cardiovascular disease, chronic kidney disease, cystatin C, GFR

## Abstract

**Background:**

In this secondary analysis of the STOP-ACEi trial, we explored the impact of discontinuing or continuing renin–angiotensin system inhibitor therapy in people with advanced chronic kidney disease on cystatin C estimated glomerular filtration rate (eGFR).

**Methods:**

Cystatin C eGFRs were calculated at baseline, 12, 24 and 36 months using Chronic Kidney Disease Epidemiology Collaboration (CKD-EPI) cystatin 2012, European Kidney Function Consortium and CKD-EPI Combined 2021 equations. We excluded samples obtained after the initiation of kidney replacement therapy. Primary analysis used complete case analysis and mixed-effects linear regression model, adjusting for minimization variables, baseline value, time-point and treatment by time interaction. Sensitivity analysis was conducted using a pattern mixture model to account for missing data that was not at random. To model the longitudinal cystatin C data with time-to-event data, a joint model was utilized which incorporated the cystatin C measurements at various time points and accounted for the occurrence of kidney replacement therapy.

**Results:**

The mean cystatin C eGFRs (CKD-EPI 2012) at baseline were 17.8 mg/L [standard deviation (SD 6.3)] and 17.9 mL/min/1.73 m^2^ (SD 6.3) in the STOP and CONTINUE arms, respectively. The estimated least squares mean difference at 12 months between STOP and CONTINUE arm was –1.46 [95% confidence interval (CI) –2.39 to –0.52, *P *= .002]. The estimated least squares mean difference at 24 months was –2.27 (95% CI –3.48 to –1.06, *P *< .001). The estimated least squares mean difference at 36 months was –1.72 (95% CI –3.48 to 0.03, *P *= .05).

**Conclusion:**

Our results are consistent with the primary study's analysis and sensitivity analyses support these findings and provide additional insights. Our findings demonstrate the similarity of creatinine and cystatin eGFR results and therefore support the use of cystatin C as an alternative marker of eGFR in advanced CKD, particularly in those in whom creatinine is likely to be less accurate.

KEY LEARNING POINTS **What was known:**

- Renin–angiotensin system (RAS) inhibitors affect cystatin C levels, a marker of kidney function.
- Cystatin C may outperform creatinine in assessing true glomerular filtration rate (GFR).
- Despite its potential in predicting cardiovascular risk and CKD progression, cystatin C is underutilized.

**This study adds:**

- Discontinuing RAS inhibitors in advanced CKD patients led to increased cystatin C levels compared with continuing therapy.
- The rise in cystatin C levels did not show sustained differences in estimated GFR (eGFRcys) between treatment arms.
- Highlights the importance of cystatin C in CKD management, urging further research on its clinical implications and the impact of RAS inhibition.

**Potential impact:**

- Encourages consideration of cystatin C measurement alongside creatinine for improved CKD assessment.
- Raises awareness about the potential benefits of targeting cystatin C levels in RAS inhibitor therapy for delaying kidney failure.
- Promotes future research to elucidate the clinical significance of cystatin C and optimise CKD management strategies.

## INTRODUCTION

In this secondary analysis of a pre-specified secondary outcome of people enrolled in the STOP-ACEi trial [1, 2], we explored the impact of discontinuing (STOP) or continuing renin–angiotensin system (RAS) inhibitor therapy in people with advanced chronic kidney disease (CKD) on serum cystatin C levels and associated estimated glomerular filtration rate (eGFR_cys_), and compared this with both the American CKD Epidemiology Collaboration (CKD-EPI) and European Kidney Function Consortium (EKFC) equations for measuring eGFR.

Cystatin C is a low molecular weight protein with chemical properties that give it value as an endogenous marker of kidney function. It is produced at a constant rate by all nucleated cells, is relatively freely filtered by glomeruli, reabsorbed and catabolized by proximal renal tubular cells, and is unaffected by muscle mass, in contrast to creatinine [3]. Studies have shown cystatin C to outperform creatinine as a marker of true kidney function and it has been demonstrated to have an increased sensitivity and accuracy for changes in true GFR [4], specifically at higher eGFR values.

In the UK, the National Institute for Health and Care Excellence (NICE) and also worldwide the Kidney Disease: Improving Global Outcomes (KDIGO) guidelines on the diagnosis, monitoring and management of CKD do not recommend routine cystatin C measurement in everyone. They do, however, endorse confirmatory testing in specific circumstances when the eGFR calculated using creatinine (eGFR_creat_) is less accurate. This may be for certain people during drug dosing or in those with extremes of muscle mass [5, 6]. eGFR using cystatin C levels has been shown to have a stronger correlation with future risk of cardiovascular disease and death than eGFR_creat_. Despite this, there is inadequate adoption of concordance testing for CKD using cystatin C, as recommended by NICE and KDIGO [5, 7].

The use of renin–angiotensin system (RAS) inhibitors including angiotensin-converting enzyme (ACE) inhibitors and angiotensin-receptor blockers (ARB) has been shown to reduce cystatin C levels in a small number of studies [8–12]. These drugs reduce blood pressure, slow the decline in eGFR and reduce proteinuria. In people with CKD, serum cystatin C levels have been shown to be a more reliable predictor of cardiovascular outcomes and risk of progression to end-stage kidney disease (ESKD) than serum creatinine, as well as identifying more people who are at increased risk such as elderly and non-white ethnic groups [13–17].

The primary outcome from the STOP-ACEi trial was the eGFR_creat_ at 3 years as calculated according to the Modification of Diet in Renal Disease (MDRD175) four-variable equation. Sensitivity analysis was also conducted with CKD-EPI 2009 equation. Data for the primary analysis were censored at the initiation of kidney replacement therapy (KRT; dialysis or transplantation).

## MATERIALS AND METHODS

In this multi-centre, open-label trial, people with advanced chronic kidney disease (eGFR <30 mL/min/1.73 m^2^) were randomly assigned to either STOP or CONTINUE RAS inhibitor therapy. Cystatin C values were obtained at baseline, 12, 24 and 36 months. Samples that were obtained after the initiation of KRT were excluded from our analysis. eGFR was calculated and compared using six different equations: CKD-EPI 2014 (cystatin), CKD-EPI 2021 (creatinine, combined) and EKFC (cystatin, creatinine, combined).

### Analytical methods

All study samples for cystatin C were analysed, centrally on a Beckman Coulter AU5820 analyser, using Gentian Cystatin C Reagent REF B08179 (Gentian AS, Bjornasveien Norway). Imprecision within batch and between batch at 0.90, 2.91 and 5.29 mg/L were 0.82%, 1.78%; 0.81%, 2.26% and 0.49%, 2.05%, respectively. The assay principle is a particle-enhanced turbidometric immunoassay (PETIA). Samples for serum creatinine were analysed at each individual investigation site.

### Statistical analysis

We used a repeated-measures, mixed-effects linear regression model (which included a term for the interaction of the time with treatment group) to estimate the between-group difference in cystatin C at 12, 24 and 36 months. A compound symmetry covariance structure was assumed. All analysis were adjusted for the minimization variables and baseline cystatin C value by including them as covariates in the model. The CONTINUE arm was the reference category for all analysis.

For complete case analysis of all four equations as well as the original blood cystatin C levels, any values post-KRT were removed from analysis since it included the added benefit of dialysis or transplant. In the first instance we did a complete case analysis for all equations and no missing data were imputed in this analysis.

To examine the effect of excluding these data that were considered missing not at random, we performed sensitivity analyses by fitting a pattern mixture model and joint model. The pattern mixture model involved a two-stage procedure using multiple imputation methods and then for those participants who initiated KRT any values post this point were replaced with their last cystatin C value prior to starting KRT. We also jointly modelled the longitudinal cystatin C data with time-to-event data for the occurrence of KRT or ESKD.

This statistical analysis aligns with the original STOPACE trial and is tailored to precisely model values, accounting for anticipated missing data commonly encountered in clinically advanced CKD patients (many of whom may experience mortality, undergo transplantation or commence dialysis) by employing the method of last observed value carried forward.

## RESULTS

### eGFR

#### CKD-EPI cystatin C 2012

**Figure 1:**
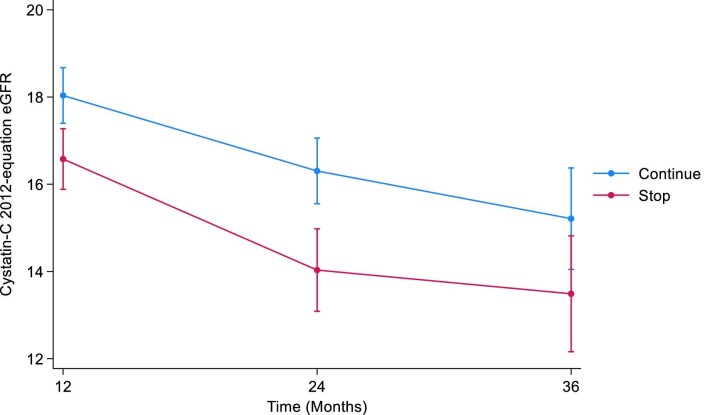
Illustrates the marginal means of CKD-EPI cystatin C 2012 eGFR over time by treatment arm.

The mean cystatin C eGFRs at baseline were 17.8 mL/min/1.73 m^2^ [standard deviation (SD 6.3)] and 17.9 mL/min/1.73 m^2^ (SD 6.3) in the STOP and CONTINUE arms, respectively. At 12 months, the mean eGFRs were 16.4 mL/min/1.73 m^2^ (SD 6.0) in the STOP arm and 17.3 mL/min/1.73 m^2^ (SD 6.1) in the CONTINUE arm. The estimated least squares mean difference at 12 months between STOP and CONTINUE arm was –1.46 mL/min/1.73 m^2^ [95% confidence interval (CI) –2.39 to –0.52, *P *= .002]. The mean cystatin C eGFRs at 24 months were 15.7 mL/min/1.73 m^2^ (SD 7.2) in the STOP arm and 16.0 mL/min/1.73 m^2^ (SD 5.1) in the CONTINUE arm. The estimated least squares mean difference at 24 months was –2.27 mL/min/1.73 m^2^ (95% CI –3.48 to –1.06, *P *< .001). At 36 months, the mean cystatin C eGFRs were 16.4 mL/min/1.73 m^2^ (SD 7.5) in the STOP arm and 16.3 mL/min/1.73 m^2^ (SD 6.6) in the CONTINUE arm. The estimated least squares mean difference at 36 months was –1.72 mL/min/1.73 m^2^ (95% CI –3.48 to 0.03, *P *= .05) (Table 1, Fig. 1). The treatment by time interaction term was *P *= .3.

**Table 1:** Complete case analysis adjusted for minimization variables, baseline value, time-point and treatment by time interaction using CKD-EPI cystatin C 2012 equation. A compound symmetry covariance structure is assumed in the model and robust standard errors used. CONTINUE arm used as reference category in the model and values <0 indicate better outcomes for STOP arm. Values after commencing KRT (i.e. dialysis/transplant) are not included in the analysis.

					Mixed-effects linear regression	
CKD-EPI cystatin C 2012	Time point	Summary statistic	STOP	CONTINUE	Mean Diff (95% CI); *P*-value	Treatment by time interaction *P*-value
CKD-EPI cystatin C 2012 (mL/min/1.73 m^2^)	Baseline	*N*	183	181		*P *= .3
		Mean (SD)	17.8 (6.3)	17.9 (6.3)		
		Min–Max	4.5–45.1	3.9–50.8		
	12 months	*N*	108	114	–1.46 (–2.39, –0.52); *P *= .002	
		Mean (SD)	16.4 (6.0)	17.3 (6.1)		
		Min–Max	3.3–44.9	3.7–33.2		
	24 months	*N*	65	70	–2.27 (–3.48, –1.06); *P *< .001	
		Mean (SD)	15.7 (7.2)	16.0 (5.1)		
		Min–Max	3.4–44.4	4.3–30.8		
	36 months	*N*	31	35	–1.72 (–3.48, 0.03); *P *= .05	
		Mean (SD)	16.4 (7.5)	16.3 (6.6)		
		Min–Max	4.8–37.7	4.5–29.4		

#### EKFC cystatin C

**Figure 2:**
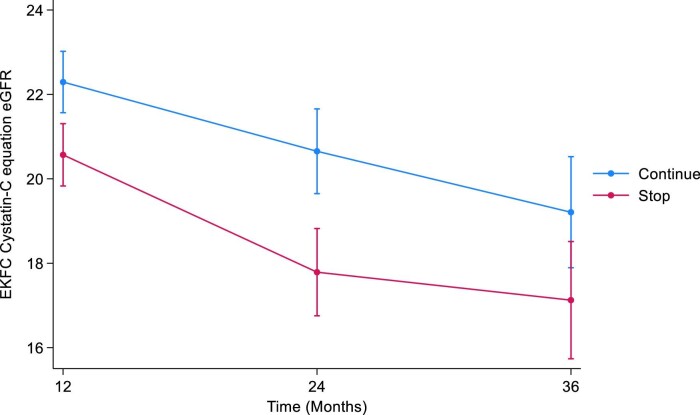
Illustrates the marginal means of EKFC eGFR over time by treatment arm.

The mean cystatin C eGFRs at baseline were 22.0 mL/min/1.73 m^2^ (SD 6.8) and 22.1 mL/min/1.73 m^2^ (SD 6.6) in the STOP and CONTINUE arms, respectively. At 12 months, the mean cystatin C eGFRs were 20.5 mL/min/1.73 m^2^ (SD 6.4) in the STOP arm and 21.4 mL/min/1.73 m^2^ (SD 6.5) in the CONTINUE arm. The estimated least squares mean difference at 12 months between STOP and CONTINUE arm was –1.73 mL/min/1.73 m^2^ (95% CI –2.77 to –0.68, *P *= .001). The mean cystatin C eGFRs at 24 months were 19.6 mL/min/1.73 m^2^ (SD 7.8) in the STOP arm and 20.3 mL/min/1.73 m^2^ (SD 5.9) in the CONTINUE arm. The estimated least squares mean difference at 24 months was –2.86 mL/min/1.73 m^2^ (95% CI –4.32 to –1.41, *P *< .001). At 36 months, the mean cystatin C eGFRs were 20.4 mL/min/1.73 m^2^ (SD 7.7) in the STOP arm and 20.2 mL/min/1.73 m^2^ (SD 7.2) in the CONTINUE arm. The estimated least squares mean difference at 36 months –2.08 mL/min/1.73 m^2^ (95% CI –3.99 to –0.18, *P *= .03) (Table 2, Fig. 2). The treatment by time interaction term was *P *= .2.

**Table 2:** Complete case analysis adjusted for minimization variables, baseline value, time-point and treatment by time interaction using EKFC equation.

					Mixed-effects linear regression	
EKFC cystatin C	Time point	Summary statistic	STOP	CONTINUE	Mean Diff (95% CI); *P*-value	Treatment by time interaction *P*-value
EKFC cystatin C (mL/min/1.73 m^2^)	Baseline	*N*	183	181		*P *= .2
		Mean (SD)	22 (6.8)	22.1 (6.6)		
		Min–Max	6.9–50.3	6.0–52.8		
	12 months	*N*	108	116	–1.73 (–2.77, –0.68); *P *= .001	
		Mean (SD)	20.5 (6.4)	21.4 (6.5)		
		Min–Max	5.4–50.3	6.0–39.1		
	24 months	*N*	65	72	–2.86 (–4.32, –1.41); *P *< .001	
		Mean (SD)	19.6 (7.8)	20.3 (5.9)		
		Min–Max	5.5–50.0	6.9–41.0		
	36 months	*N*	31	35	–2.08 (–3.99, –0.18); *P *= .03	
		Mean (SD)	20.4 (7.7)	20.2 (7.2)		
		Min–Max	7.0–40.7	6.9–34.1		

#### CKD-EPI combined creatinine–cystatin C 2021

**Figure 3:**
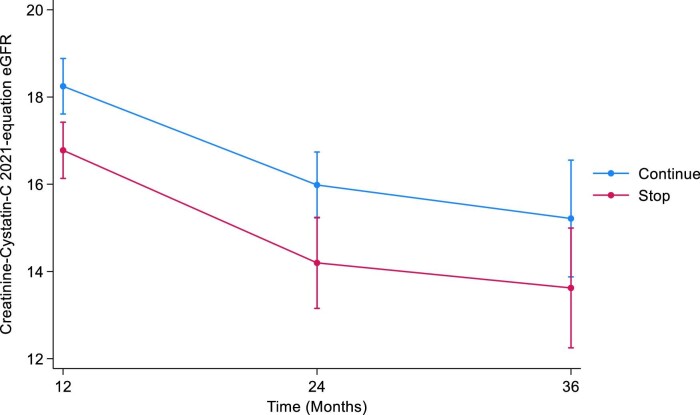
Illustrates the marginal means of CKD-EPI combined 2021 eGFR over time by treatment arm.

The mean combined eGFR at baseline were 17.8 mL/min/1.73 m^2^ (SD 5.8) and 17.9 mL/min/1.73 m^2^ (SD 5.5) in the STOP and CONTINUE arms, respectively. At 12 months, the mean combined eGFRs were 16.3 mL/min/1.73 m^2^ (SD 5.7) in the STOP arm and 17.3 mL/min/1.73 m^2^ (SD 6.1) in the CONTINUE arm. The estimated least squares mean difference at 12 months between STOP and CONTINUE arm was –1.47 mL/min/1.73 m^2^ (95% CI –2.36 to –0.58, *P *= .001). The mean combined eGFR at 24 months were 15.8 mL/min/1.73 m^2^ (SD 6.9) in the STOP arm and 16.0 mL/min/1.73 m^2^ (SD 4.8) in the CONTINUE arm. The estimated least squares mean difference at 24 months was –1.79 mL/min/1.73 m^2^ (95% CI –3.07 to –0.50, *P *= 0.01). At 36 months, the mean combined eGFRs were 17.1 mL/min/1.73 m^2^ (SD 7.8) in the STOP arm and 16.8 mL/min/1.73 m^2^ (SD 6.9) in the CONTINUE arm. The estimated least squares mean difference at 36 months was –1.59 mL/min/1.73 m^2^ (95% CI –3.51 to 0.32, *P *= .1) (Table 3, Fig. 3). The treatment by time interaction term was *P *= .8.

**Table 3:** Complete case analysis adjusted for minimization variables, baseline value, time-point and treatment by time interaction using CKD-EPI combined 2021 eGFR equation.

					Mixed-effects linear regression	
CKD-EPI combined 2021	Time point	Summary statistic	STOP	CONTINUE	Mean Diff (95% CI); *P*-value	Treatment by time interaction *P*-value
CKD-EPI combined 2021 (mL/min/1.73 m^2^)	Baseline	*N*	183	181		*P *= .8
		Mean (SD)	17.8 (5.8)	17.9 (5.5)		
		Min–Max	6.4–41.9	5.2–35.1		
	12 months	*N*	107	113	–1.47 (–2.36, –0.58); *P *= .001	
		Mean (SD)	16.3 (5.7)	17.3 (6.1)		
		Min–Max	4.1–38.5	5.0–32.0		
	24 months	*N*	64	70	–1.79 (–3.07, –0.50); *P *= .01	
		Mean (SD)	15.8 (6.9)	16.0 (4.8)		
		Min–Max	3.8–36.3	6.2–29.2		
	36 months	*N*	31	35	–1.59 (–3.51, 0.32); *P *= .1	
		Mean (SD)	17.1 (7.8)	16.8 (6.9)		
		Min–Max	6.3–39.8	5.8–35.6		

### Sensitivity analysis—pattern mixture model

#### CKD-EPI cystatin C 2012

**Figure 4:**
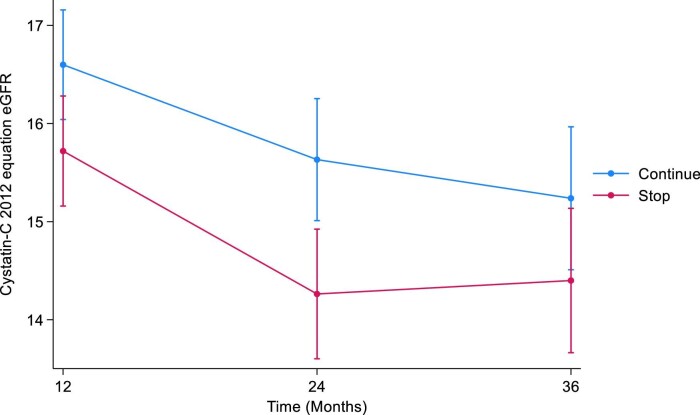
Displays the marginal means of CKD-EPI cystatin 2012 eGFR over time by treatment arm using the pattern mixture model with LOCF imputation. LOCF, last observation carried forward.

At 12 months, the estimated least squares mean difference in cystatin C eGFR between the STOP and CONTINUE arms was –0.88 (95% CI –1.70 to –0.06, *P *= .04). At 24 months, the estimated least squares mean difference in cystatin C eGFR was –1.37 (95% CI –2.29 to –0.45, *P *= .004). At 36 months, the estimated least squares mean difference in cystatin C eGFR was –0.84 (95% CI –1.88 to 0.20, *P *= .1) (Table 4, Fig. 4). The treatment by time interaction term was *P *= .05.

**Table 4:** Pattern mixture model sensitivity analysis estimated least squares mean difference in CKD-EPI cystatin C 2012 eGFR between the STOP and CONTINUE arms.

		Mixed-effects linear regression	
Pattern mixture model (LOCF imputed)	Time point	Mean Diff (95% CI); *P*-value	Treatment by time interaction *P*-value
CKD-EPI cystatin C 2012 (mL/min/1.73 m^2^)	12 months	–0.88 (–1.70, –0.06); *P *= .04	*P *= .5
	24 months	–1.37 (–2.29, –0.45); *P *= .004	
	36 months	–0.84 (–1.88, 0.20); *P *= .1	

LOCF, last observation carried forward.

#### EKFC cystatin C

**Figure 5:**
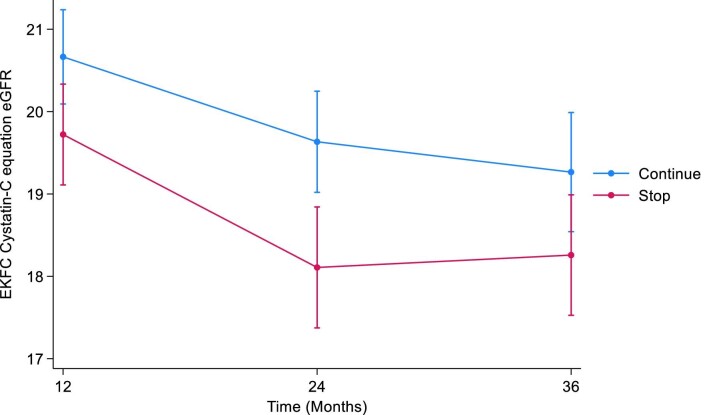
Displays the marginal means of EKFC eGFR over time by treatment arm using the pattern mixture model with LOCF imputation. LOCF, last observation carried forward.

At 12 months, the estimated least squares mean difference in cystatin C eGFR between the STOP and CONTINUE arms was –0.94 (95% CI –1.80 to –0.09, *P *= .03). At 24 months, the estimated least squares mean difference in cystatin C eGFR was –1.53 (95% CI –2.45 to –0.60, *P *= .001). At 36 months, the estimated least squares mean difference in cystatin C eGFR was –1.01 (95% CI –2.02 to 0.00, *P *= .05) (Table 5, Fig. 5). The treatment by time interaction term was *P *= .4.

**Table 5:** Pattern mixture model sensitivity analysis estimated least squares mean difference in EKFC eGFR between the STOP and CONTINUE arms.

		Mixed-effects linear regression	
Pattern mixture model (LOCF imputed)	Time point	Mean Diff (95% CI); *P*-value	Treatment by time interaction *P*-value
EKFC cystatin C (mL/min/1.73 m^2^)	12 months	–0.94 (–1.80, –0.09); *P *= .03	*P *= .4
	24 months	–1.53 (–2.45, –0.60); *P *= .001	
	36 months	–1.01 (–2.02, 0.00); *P *= .05	

LOCF, last observation carried forward.

#### CKD-EPI combined creatinine–cystatin C 2021

**Figure 6:**
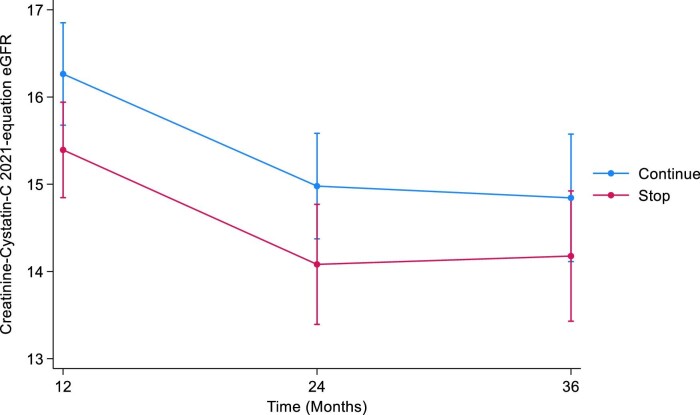
Displays the marginal means of CKD-EPI combined 2021 eGFR over time by treatment arm using the pattern mixture model with LOCF imputation. LOCF, last observation carried forward.

At 12 months, the estimated least squares mean difference in combined eGFR between the STOP and CONTINUE arms was –0.87 (95% CI –1.66 to –0.08, *P *= .03). At 24 months, the estimated least squares mean difference in combined eGFR was –0.90 (95% CI –1.81 to 0.02, *P *= .05). At 36 months, the estimated least squares mean difference in combined eGFR was –0.67 (95% CI –1.71 to 0.37, *P *= .2) (Table 6, Fig. 6). The treatment by time interaction term was *P *= .9.

**Table 6:** Pattern mixture model sensitivity analysis estimated least squares mean difference in CKD-EPI Combined 2021 eGFR between the STOP and CONTINUE arms.

		Mixed-effects linear regression	
Pattern mixture model (LOCF imputed)	Time point	Mean Diff (95% CI); *P*-value	Treatment by time interaction *P*-value
CKD-EPI combined 2021 (mL/min/1.73 m^2^)	12 months	–0.87 (–1.66, –0.08); *P *= .03	*P *= .9
	24 months	–0.90 (–1.81, 0.02); *P *= .05	
	36 months	–0.67 (–1.71, 0.37); *P *= .2	

LOCF, last observation carried forward.

### Sensitivity analysis—joint model

#### CKD-EPI cystatin C 2012

**Figure 7:**
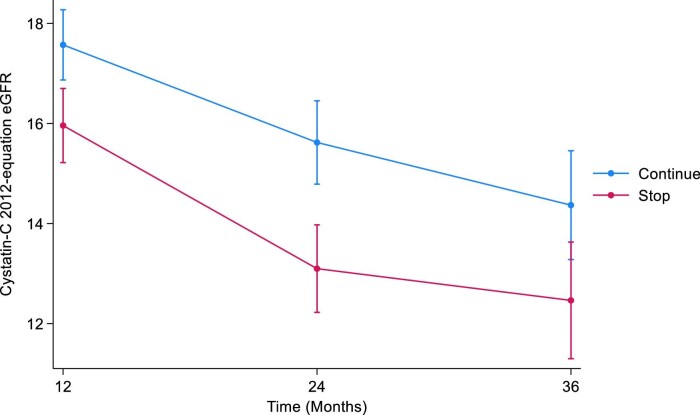
Marginal means of CKD-EPI cystatin 2012 eGFR over time by treatment arm using the joint model with LOCF imputation. LOCF, last observation carried forward.

The estimated least squares mean difference in cystatin C eGFR between the STOP and CONTINUE arms was –1.61 at 12 months (95% CI –2.61 to –0.61, *P *= .002). At 24 months, the estimated least squares mean difference in cystatin C eGFR was –2.52 (95% CI –3.71 to –1.34, *P *< .001). The estimated least squares mean difference in cystatin C eGFR at 36 months was –1.90 (95% CI –3.46 to –0.34, *P *= .02) (Table 7, Fig. 7). The treatment by time interaction term was *P *= .3.

**Table 7:** : Joint model sensitivity analysis estimated least squares mean difference in CKD-EPI cystatin C 2012 eGFR between the STOP and CONTINUE arms.

Joint model	Time point	Estimated Mean Diff (95% CI); *P*-value	Treatment by time interaction *P*-value
CKD-EPI cystatin C 2012 (mL/min/1.73 m^2^)	12 months	–1.61 (–2.61, –0.61); *P *= .002	*P *= .3
	24 months	–2.52 (–3.71, –1.34); *P *< .001	
	36 months	–1.90 (–3.46, –0.34); *P *= .02	

#### EKFC cystatin C

**Figure 8:**
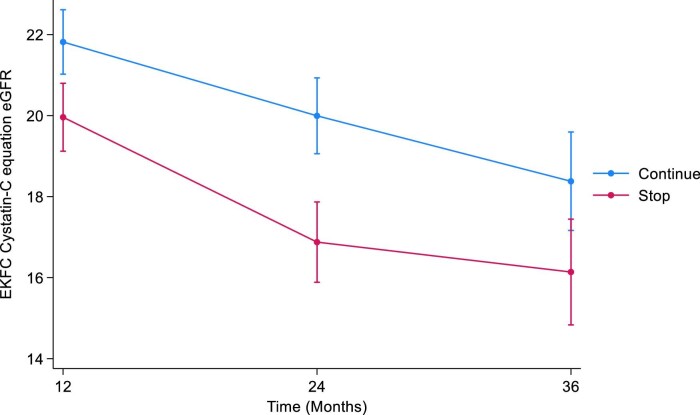
Marginal means of EKFC eGFR over time by treatment arm using the joint model with LOCF imputation. LOCF, last observation carried forward.

The estimated least squares mean difference in cystatin C eGFR between the STOP and CONTINUE arms was –1.86 at 12 months (95% CI –2.99 to –0.72, *P *= .001). At 24 months, the estimated least squares mean difference in cystatin C eGFR was –3.12 (95% CI –4.45 to –1.78, *P *< .001). The estimated least squares mean difference in cystatin C eGFR at 36 months was –2.24 (95% CI –3.98 to –0.49, *P *= .01) (Table 8, Fig. 8). The treatment by time interaction term was *P *= .1.

**Table 8:** Joint model sensitivity analysis estimated least squares mean difference in EKFC eGFR between the STOP and CONTINUE arms.

Joint model	Time point	Estimated Mean Diff (95% CI); *P*-value	Treatment by time interaction *P*-value
EKFC cystatin C (mL/min/1.73 m^2^)	12 months	–1.86 (–2.99, –0.72); *P *= .001	*P *= .1
	24 months	–3.12 (–4.45, –1.78); *P *< .001	
	36 months	–2.24 (–3.98, –0.49); *P *= .01	

#### CKD-EPI combined creatinine–cystatin C 2021

**Figure 9:**
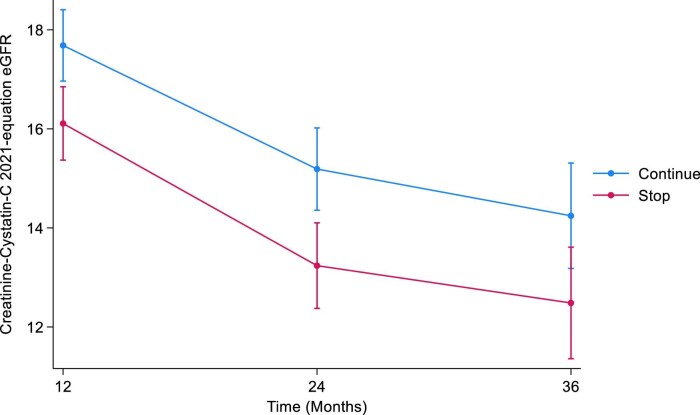
Marginal means of CKD-EPI combined 2021 eGFR over time by treatment arm using the joint model with LOCF imputation. LOCF, last observation carried forward.

The estimated least squares mean difference in combined eGFR between the STOP and CONTINUE arms was –1.58 at 12 months (95% CI –2.58 to –0.57, *P *= .002). At 24 months, the estimated least squares mean difference in combined eGFR was –1.95 (95% CI –3.12 to –0.78, *P *= .001). The estimated least squares mean difference in combined eGFR at 36 months was –1.76 (95% CI –3.27 to –0.25, *P *= .02) (Table 9, Fig. 9). The treatment by time interaction term was *P *= .8.

**Table 9:** Joint model sensitivity analysis estimated least squares mean difference in CKD-EPI combined 2021 eGFR between the STOP and CONTINUE arms.

Joint model	Time point	Estimated Mean Diff (95% CI); *P*-value	Treatment by time interaction *P*-value
CKD-EPI combined 2021 (mL/min/1.73 m^2^)	12 months	–1.58 (–2.58, –0.57); *P *= .002	*P *= .8
	24 months	–1.95 (–3.12, –0.78); *P *= .001	
	36 months	–1.76 (–3.27, –0.25); *P *= .02	

### Creatinine and cystatin comparison

**Figure 10:**
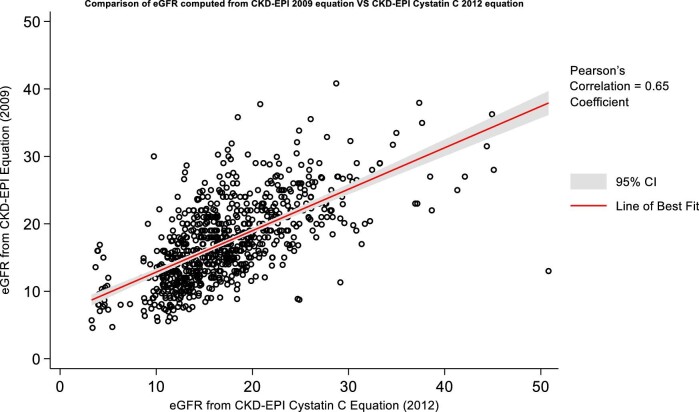
Scatter plot of the eGFR values computed from CKD-EPI 2012 cystatin and CKD-EPI 2009 creatinine eGFR equations with a line of best fit and 95% CIs. Calculated Pearson's correlation coefficient has a value of 0.65.

The scatter plot (Fig. 10) compares eGFR values derived from the CKD-EPI 2012 cystatin C equation with those from the CKD-EPI 2009 creatinine equation. The Pearson correlation coefficient for these eGFR values is 0.65, reflecting a substantial positive correlation between the two equations.

## DISCUSSION

Sensitivity analyses using pattern mixture and joint models produced results consistent with the primary analysis. The STOP arm exhibited higher cystatin C eGFR values compared with the CONTINUE arm, with significant differences observed at various time points. However, while some analyses showed statistical significance favouring the CONTINUE arm, these findings were insufficient to establish clinical superiority.

**Figure 11:**
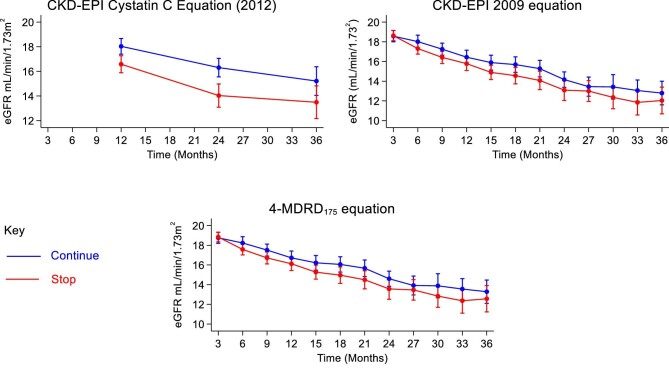
Combined graphs showing the eGFR values from the final fitted model (based on complete case analysis) for the CKD-EPI 2012 cystatin C, CKD-EPI 2009 creatinine and four-variable MDRD175 creatinine eGFR equations.

A side-by-side comparison of CKD-EPI 2012 cystatin C, CKD-EPI 2009 creatinine and MDRD175 eGFR (primary outcome of main study) reveals their similarity (Fig. 11). A scatter plot of eGFR values (Fig. 10) calculated from the cystatin C–based CKD-EPI 2012 equation and the CKD-EPI 2009 equation, which includes a line of best fit with a 95% CI, revealed a Pearson correlation coefficient of 0.65. This coefficient indicates a strong positive correlation between the two equations, suggesting that cystatin C is a reliable alternative for eGFR measurement in the absence of creatinine.

The limitation in establishing clinical superiority arises from the design of the primary trial, which was based on the MDRD eGFR measure at the 3-year time point and used a minimum clinically important difference of 5 points for sample size calculations. The cystatin C analyses did not detect a 5-point difference at any time point, including the primary 3-year assessment. As a result, although statistically significant findings were noted, they are unlikely to be clinically meaningful. Additionally, the cystatin C results involved multiple equations and time points, which warrants caution in interpreting significant findings due to potential multiple testing issues.

ACE inhibitors and ARBs are widely prescribed for the management of hypertension, ischaemic heart disease and heart failure, which are significant factors contributing to cardiovascular morbidity and mortality in people with kidney disease. Both ACE inhibitors and ARBs are known to have reno-protective effects as a class. However, there is limited research available on the impact of ACE inhibitors or ARBs on serum cystatin C levels [8–11]. In a study conducted by Watanabe *et al*. in 30 patients without diabetes but with hypertension who were administered valsartan for a duration of 3 months [12], the authors found a significant reduction in serum cystatin C levels, suggesting that the treatment with valsartan could potentially decrease renal vascular resistance in hypertensive patients and potentially prevent future kidney failure.

There is a growing body of evidence suggesting that the use of cystatin C is valuable in both diagnosing and managing CKD. Compared with serum creatinine, cystatin C is more precise in detecting initial stages of CKD and in predicting adverse outcomes such as kidney failure, cardiovascular disease and mortality. A recent comprehensive study of 400 000 participants conducted within the UK Biobank revealed that incorporating both eGFR_cys_ and eGFR_creat-cys_ alongside traditional risk factors for atherosclerotic diseases significantly improved the accuracy of predictions for overall mortality, as well as fatal and non-fatal cardiovascular disease [13, 14]. Interestingly, the inclusion of eGFR_cys_ demonstrated the most significant enhancement in distinguishing these risks, whereas the addition of eGFR based on creatinine did not contribute to improved discrimination.

In an analysis conducted on over 11 000 participants from the Multi-Ethnic Study of Atherosclerosis (MESA) and Cardiovascular Health Study (CHS) in 2011, hazard ratios for mortality were examined among individuals with and without CKD. The analysis considered adjusted hazard ratios for different CKD identification methods [16]. In the MESA study, the hazard ratio for mortality was 0.80 for CKD identified by creatinine, 3.23 for CKD identified by cystatin C and 1.93 for CKD identified by both markers. Similarly, in the CHS study, the adjusted hazard ratios were 1.09, 1.78 and 1.74, respectively. This consistent pattern was observed across various outcomes, including cardiovascular disease, heart failure and kidney failure. Notably, individuals identified as having CKD based on the cystatin C–based equation exhibited an unfavourable prognosis.

Initiating an ACE inhibitor reduces angiotensin II levels which leads to a decrease in efferent arteriolar pressure. Consequently, this reduction in pressure lowers intraglomerular pressure by decreasing transcapillary hydrostatic pressure along the glomerular capillary. Additionally, ACE inhibitors cause a slight decrease in the tubular secretion of creatinine into the urinary space and an enhancement in proximal tubular reabsorption. These mechanisms contribute to the predictable increase of approximately 15%–30% in serum creatinine levels observed in patients using drugs that antagonize the RAS. Given that creatinine is significantly smaller than cystatin C, it is more easily filtered across the glomerular barrier into the urinary space. Therefore, a drop in glomerular pressure resulting from RAS inhibitors leads to reduced filtration of creatinine. In contrast, serum cystatin C reaches a steady state in the blood and is less responsive to fluctuations in intraglomerular pressure, meaning it may not change significantly with the introduction of a RAS inhibitor.

## CONCLUSION

This analysis of the STOP-ACEi Trial provides additional insights into the effects of discontinuing or continuing RAS inhibitor therapy in patients with advanced CKD on cystatin C eGFR. The results demonstrate consistency with the findings of the main trial results, showing no clinically relevant change in the eGFR_cys_. Differences in cystatin C eGFR between treatment arms at 24 months were not persistent at 36 months, nor likely to be clinically significant.

Our results are consistent with the primary study's analysis and sensitivity analyses support these findings and provide additional insights. These findings contribute to the growing body of evidence supporting the use of cystatin C as an important alternative marker for diagnosing, monitoring and managing CKD. Although not specifically analysed in this study, we can conclude that this paper does support the use of cystatin C in specific populations where creatinine is known to be less reliable, such as the elderly, sarcopenic patients or patients at extremes of muscle mass.

Cystatin C has been shown to have greater accuracy than creatinine in detecting initial stages of CKD and predicting adverse outcomes such as kidney failure and cardiovascular disease. The findings from this analysis suggest that calculating cystatin C eGFR may have value in advanced CKD but did not identify a clinically relevant change in eGFR_cys_ versus other eGFR calculations. The impact of RAS inhibition on cystatin C levels may be a valuable research target with a potential benefit in delaying future kidney failure, which will need further dedicated studies to explore.

Overall, this analysis supports the findings of the main STOP-ACEi trial and raises questions regarding cystatin C as a potential treatment target for people with CKD. Further studies are warranted to explore the clinical implications of cystatin C measurement and the long-term effects of RAS inhibitor therapy on kidney and cardiovascular outcomes in people with CKD.

## Data Availability

The data supporting the findings of this study and summary statistics are available by request by contacting supervising author S.B. at sunil.bhandari@nhs.net.
